# The Hospital Nursing Supplement

**Published:** 1896-02-01

**Authors:** 


### "The Hospital" Nursing Mirror.

Being the Extra Nursing Supplement of "The Hospital" Newspaper.

[Contributions for this Supplement should be addressed to the Editor, The Hospital, 428, Strand, London, W.C., and should have the word "Nursing" plainly written in left-hand top corner of the envelope.]

### News from the Nursing World.

### OUR COURTEOUS QUEEN.

Amongst the numerous messages of condolence which reached Osborne last week there was a modest little letter from a nurse. She ventured to assure Her Majesty of the deep sympathy felt by all nurses with the Queen and Princess Beatrice in their great sorrow. A reply to this note was hardly expected, but it came immediately in the form of a telegram from a member of the Royal household, "Commanded to thank you for your sympathetic letter."

### THE NURSES' CO-OPERATION.

Nurses who devote themselves to private nursing have often been cautioned against engaging themselves to so-called institutions of which they have no accurate knowledge. Our letter-box reveals from time to time the serious straits, often due to ill-usage, which innocent nurses are reduced to by the conductors of these private venture institutions, which exist, in fact, to make large profits for the proprietors at the expense of everybody else. Neither the public nor the nurses are fairly treated by the conductors of these so-called nursing institutions. The reason for their increase in late years may be indirectly gathered from the progress of the Nurses' Co-operation, 8, New Cavendish Street, W. This co-operation is, we believe, the only institution which has ever been founded with the sole aim of securing to the nurses all their earnings without deduction of any kind. Voluntarily the nurses agreed to pay 7½ per cent. for a brief period, and subsequently 5 per cent. only on their earnings towards the expenses of conducting the Co-operation. In three years this 5 per cent. has not only yielded enough to pay all the expenses, but has enabled the Co-operation to accumulate a capital sum as a reserve fund amounting to upwards of £2,000. The nurses of the Co-operation earned altogether £27,471 in 1895. If private nurses as a body would take these figures to heart, and combine to extend the Nurses' Co-operation, their condition would be much more comfortable and their earnings considerably larger than at present. It is time that every nurse realised that she belongs to a calling which is being exploited by many people for their own benefit at the cost of the nurses and of the public. Our columns afford eloquent testimony to this fact, as those who study current events must realise if they will pause to give their best thought to the principles which underlie many controversies now engaging the attention of the nursing world. The public, too, should combine in their own interest to support the Nurses' Co-operation, which gives them a guarantee that any nurse supplied by the Co-operation may be relied upon to give every satisfaction to the practitioner and the patient.

### WORKING HOURS AT LOCKERBIE.

The nurse employed by the Lockerbie District Nursing Association is frequently on duty from fourteen to twenty hours at a stretch, according to the recently-published annual report. The lady superintendents are nine in number, and they express themselves as "thoroughly satisfied with Nurse Hadley." The report also states that "all the officials feel sure she never spares herself, but puts her whole soul into her work." It does not appear to occur to these ladies and gentlemen that they are in any way to blame for permitting a good woman, who "never spares herself," to work for such outrageous hours at a most exhausting branch of nursing. They also countenance Nurse Hadley's undertaking the laying-out of the dead, although they own that this does not come within her range of duty. We hope that the next report issued by the Lockerbie Working Committee will show that a more humane policy has been introduced by means of an increased staff, or, at any rate, of decreased hours of work and a limited range of duties. Nurse Hadley has been employed in a large number of important and serious cases, and she must be a skilled nurse, well deserving of consideration as well as praise.

### NURSING SOLDIERS' FAMILIES.

A most encouraging report of the work of the district nurses at Aldershot was laid before the committee at the recent quarterly meeting at which Her Royal Highness the Duchess of Connaught presided. The aid given to the wives and families of the soldiers by the nurses has been highly appreciated, and their services have been invaluable to the poor women for whom illness or disablement means poverty from loss of work. The Soldiers and Sailors' Families Association has in this, as in other ways, given help of a very practical description in Aldershot, and the doctors cordially testify to the excellent work of the three nurses both in Aldershot and Farnborough. The society has received very generous support from the officers, and it will no doubt enlarge the already extensive field of its labours.

### SCRUBBERS AS ASSISTANT NURSES.

It appears from the published accounts of a recent board meeting at Islington that the infirmary nurses have scrubbers and helpers to assist them, but these persons do not "assist in surgical dressings." A Guardian proposed that the Infirmary Committee should be instructed to consider the advisability of appointing nurses in place of these assistants. This very proper suggestion was negatived on the ground of there being no suitable accommodation for more nurses, but we are glad to see by a later report that the Visiting Committee, on considering the matter, have recommended that the Local Government Board be asked to sanction the employment of twelve paid nurses in place of the helpers, and of two additional nurses for the separation wards. They also suggested that the necessary sleeping accommodation could be obtained by renting some adjoining houses, and this latter question was finally referred to the House Committee for arrangement.

### DISTRICT NURSES AT PAISLEY.

A happy party assembled in the Peter Brough Nursing Home the other day, when the nurses entertained the children they had attended in their own homes at Paisley during the last six months. After a tea, to which full justice was done by the juvenile guests, a lovely Christmas tree was displayed to their delighted eyes, and each received a toy, sweets, and fruit from the heavily-laden branches. Music and games followed, and probably every child cordially agreed with a little boy who was heard to say, "I wish it came every week!"

### COVENTRY NURSING INSTITUTION.

The annual meeting of the Coventry Nursing Institute was recently held in the Mayor's Parlour, when there was a large attendance of subscribers. The work of the district nurses continues to increase, one of the speakers saying that they were "always on duty"; an addition to the staff is desirable as soon as ever funds are forthcoming. The private nursing branch of the institute, which is a distinct one, appears to work satisfactorily, and it was pleasant to hear the just view respecting nurses' earnings which exists at Coventry, for it was acknowledged in the course of the meeting that the ideal form of business in regard to nursing was that the nurses should work for themselves, and pay the institution a percentage on their earnings. Probably at some future date such a scheme may prove workable at Coventry, and in the meantime we trust increased subscriptions will be obtained for the district nursing branch of the institution.

### SUNDERLAND NURSING INSTITUTE.

The Sunderland Nursing Institute, closed in October last, in consequence of an inquiry into its management, was reopened on December 30th under the superintendence of Miss Arnott, a lady trained at the Sunderland Infirmary, who has temporarily undertaken the work of reorganising the institution in both its branches, district and private nursing. There are at present two district nurses and nine private nurses, but it is intended to gradually increase the number. The committee are anxious to secure efficient and trustworthy nurses on their staff, two years' training being required. The district nursing accounts are kept and audited separately, and for the adequate maintenance of this part of the work funds are asked from the public, with the intention—should the response be sufficiently generous—of placing it under separate management.

### WELL CARED FOR.

At St. Catherine's Home, Bradford, where advanced phthisis, cancer, and other sad cases of disease are admitted, a great deal is done to lighten the burdens of the sufferers. They are excellently nursed, and their food and surroundings are alike good and pleasant. Christmas has been made glad to them by the numerous kindnesses of friends of the Home, who spared no pains to make happy the solitary anniversary which most of them are likely to spend at St. Catherine's. Gifts for each inmate were provided by the committee and other friends, and of the concert which followed later it is recorded that "it did the poor patients good; they can talk of nothing else."

### ON THE LABRADOR COAST.

In the little hospital at Indian Harbour, established by the Mission to Deep Sea Fishermen, there are various endowed beds. That known as the Ratho Cot is supported by school boys, then there is one kept up by the Brunswick Street Methodists, and another is the St. Paul's Church Cot. Altogether nine beds are available, but all through last season the average number of in-patients exceeded the accommmodation. Not infrequently the doctor had to give up his own room and make the best of a temporary shakedown in the sitting-room, for patients brought long distances were generally too ill for their admittance to be postponed. It is proposed to enlarge this much-needed hospital as soon as possible, and the building of a little church now in progress will set free for patients one room which has hitherto been used for services.

### LES ENFANTS ASSISTÉS.

The headquarters of this great French charitable institution attract the attention of many visitors in Paris. There is much to interest in the various departments, from the first rooms where the babies are kept under observation for several days after admission, to the infirmary wards in which are nursed tall lads who, brought up under the Assistance Publique, have fallen sick in service. The pavilions for infectious cases are light and airy, and the position of the beds enables the patients to look out through the numerous windows, as well as permitting of the occupants being viewed from without. The vast chamber in which some fifty infants are in charge of sisters is a clean, orderly apartment, with rows of swinging cots. The department allotted to the older babies is not so picturesque nor so airy, being probably an older building. The playground of the bigger boys has two special taps connected with the dispensary, through which a stream of cocoa and of gentian respectively flows, the supply of the former being generally exhausted first on account of its greater popularity. It has been recently stated that there is a failure on the part of the authorities to inform mothers of the illness, and even of the death of their little ones, the first intimation reaching these poor women only when they arrive to claim their children. The hardship of such a practice must press heavily on those who have deposited a child temporarily at the institution during their own pressing straits, such as destitution or the mother's own illness. A more humane policy will doubtless be adopted in this matter now that public attention has been called to it.

### SHORT ITEMS.

The Bideford Nursing Association held its annual meeting recently, and an eloquent appeal for larger financial support was made by the Rev. Roger Granville, the rector.—A memorial signed by a large number of local ladies has been dispatched to the Home Office from Cardiff, praying for the appointment of a woman doctor at the gaol, in addition to the usual medical officer.—An insane soldier at the German Military Hospital at Mons is reported to have killed two patients and wounded three others.—The programme for the annual concert given by the students to the nursing staff at St. Thomas's Hospital was as varied and excellent as that arranged in previous years.

### Lectures on Nursing.

By a Superintendent of Nurses.

### VII.—VENTILATION.

I would now call your attention to the subject of ventilation, for certain it is that of all the things a nurse should know, nothing can be of more importance than that she should be acquainted with the laws of ventilation. It is of the utmost moment to her patient that he should breathe a pure atmosphere—as pure as the outside air—and yet that he should never be exposed to a draught. You have all probably seen a stagnant pool of water in the country, and if someone walking with you at the time began to drink of it you would be inclined to think he had suddenly gone out of his mind. Now this is precisely what we are doing when we inhale foul air, and if we could see its impurity we should be filled with alarm. Air and water are necessary for the maintenance of life. Water is taken into the system in various ways—in the fluid we drink, in the food we eat, the air we breathe, and is absorbed through the pores of the skin. People are generally very frightened of drinking water they think is impure—so much so that when travelling abroad they will rarely even taste it.

They should be equally afraid of inhaling air that is contaminated. In every room or ward you want to provide for the escape of foul and the supply of fresh air. In a former lecture I described to you the composition of dust in hospital wards. Dust being the deposit of the atmosphere, you are always liable to take into your lungs the harmful particles floating about.

Air is a mixture chiefly of two gases — nitrogen and oxygen. There are 79 volumes of nitrogen and 21 of oxygen in 100 of air. Oxygen is necessary for the maintenance of animal life.

The changes which the air undergoes by its contact with the lungs are : (1) Its temperature is raised ; (2) its oxygen is diminished ; (3) the carbonic acid and aqueous vapour are increased ; (4) a small quantity of ammonia and organic matter is added to it.

For every volume of air inspired into the lungs 4½ per cent. of oxygen is absorbed by the blood, and about the same quantity of poisonous carbonic acid gas is thrown off; this process is called the aeration of blood, and consists essentially of this interchange of these two gases. The blood is the great nourisher and purifier of the body, and the blood itself is purified by this process of aeration in the lungs. The blood flowing into the lungs is dark, venous, or impure, containing a considerable amount of carbonic acid, which is an effete product of the tissues. In the lungs, as we stated, this gas is given off, and its place taken by oxygen, oxygen rendering the blood pure or arterial, and bright red in colour. This is the blood which flows from the lungs to the heart, which then pumps it to all the tissues of the body, imparting to them the oxygen which is necessary for their nutrition and activity.

It is essential for the maintenance of life that the blood should be aerated, and if the process of aeration is stopped entirely for a few minutes death is the result. Everyone has heard of suffocation, but everyone does not know that if the blood is not purified, diseased and shortened lives must follow. You can thus understand how necessary it must be to have a constant supply of fresh and removal of vitiated air, because if we breathe over and over again the same air that has been expired from the lungs of course the proportion of carbonic acid and organic matter will go on increasing until they produce fatal results. Long before this occurs the poisoned condition of blood causes drowsiness, headache, and fainting.

It was the vitiated condition of the atmosphere in the Black Hole of Calcutta that worked such frightful havoc to the poor prisoners, causing the death of 123 out of 146 shut up in it for one night.

Each person requires 3,000 cubic feet of fresh air per hour for breathing and a room 10 feet by 10 feet by 10 feet would give the required space if the air could be changed three times in an hour.

If it is necessary to change the air breathed by persons in health, how much more so is it when the atmosphere is charged with the germs of disease.

A nurse should pay the very greatest attention to the ventilation of her wards. I admit that it is a most difficult thing to let in fresh air, and yet to avoid draughts. But remember, the wind does not blow from east and west or north and south at the same time, consequently if you find a draught when the windows are open on the east side, close them and open those on the opposite side. It often happens that patients feel a great draught and yet do not like to complain, and in such a case there is often a want of thought on the part of the nurses ; the only excuse being that they probably are so warm with having so much exercise that they do not notice the cold air. Miss Nightingale says : " For the sick, warming is a necessary part of ventilation ; a careful nurse will keep a constant watch over her sick, especially weak cases, to guard against the loss of vital heat by the patient himself."

In some diseased conditions very little heat is produced, and the call made upon the vital parts to supply the deficiency may greatly weaken them, or, indeed, prove their death. With such cases the nurse should constantly be on the watch, examining the lower extremities from time to time, and should be ready with hot drinks or hot flannels to restore warmth if she finds a tendency to chilliness. This is of the utmost importance, as it is no uncommon thing for a patient in the latter stages of disease to sink for want of this external heat.

The lowest temperature of the twenty-four hours is in the early morning, and it is then that the greatest care is required to prevent the fatal chill taking place. The vital powers are less able to resist cold in the morning. If patients are feverish at night they are sure to be chilly in the morning. To make a room cold is not, as some people imagine, to ventilate it. The safest atmosphere for a patient is that of a room where there is a nice bright fire and an open window, except in extremes of temperature. Notice for yourselves what a cold place the front of a fire is, and how cold your back becomes when you sit there. You will then form an idea of the rush of air constantly being carried up the chimney. Now, if foul air leaves the room in this way and is replaced by fresh air, you see how pure the atmosphere may be kept.

When you want a fire to burn you clear away the ashes so that air may get at the fire, and as nurses you should know how to lay a fire so that it may quickly burn up, and how to keep one burning brighly, for nothing is more depressing than to see a feeble bit of fire struggling to show itself through large lumps of coal. People sometimes say they cannot think why a fire does not burn. If you put a towel over a man's nose and mouth you will suffocate him, and in like manner a fire must breathe or it will die. Try to keep your patient in an equable temperature ; do not have a roasting fire one hour and a few red embers an hour or two afterwards. In a medical ward the temperature should never be under 63 deg., and, unless specially desired, over 66 deg. In surgical wards it should not be over 63 deg. Study the ward thermometer. Pay special attention to the ventilation of the lavatories, and try to prevent a draught or rush of cold air coming down on to the patients allowed to go there.

Think what sudden chills may be given to persons getting out of warm beds, and merely covered with a cloak, if on entering the w.c.s they meet a cold gust of wind. The invalids may not dislike it, and it may seem refreshing to them, but it is none the less dangerous. You know when you are perspiring after a hot walk you may feel glad of the nice cool draught of air, and when a kindly friend warns you, you pay no heed, because you are "so hot"; but how often have you taken cold in this way? It is your duty to think for your patients, and to make them obey you.

It may be that when you open the ventilators in the morning the wind is in the south, and you arrange accordingly; but do not forget the wind may change to the east in the course of the day, and the ventilators previously opened should be closed, and others opened. Do not be satisfied by just saying, "There is a great draught somewhere," and then think you have done your duty. Do not rest till the cause is found out and the evil remedied. Night nurses should be very careful to have a warm atmosphere whilst the beds are being made. What can be more miserable for an invalid than to sit shivering by his bedside in the early, chilly morning? If he gets back to bed cold and wretched, he may be shivering for hours, and may not even know of the existence of a hot bottle. Should you find a patient in this condition, pray let him have a hot tin to his feet, and he will then probably soon fall asleep on getting back to bed.

### Nursing in Florence.

### III.—SPEDALE DEGLI INNOCENTI (FOUNDLING HOSPITAL).

This ancient building, which was commenced in 1419 and opened in 1451, forms one of the sides of the Piazza which contains the beautiful church of Saint Annunziata, and has on the façade the well-known blue medallions with the figure in white of a baby in swaddling clothes.

The old-fashioned turnstile or ruota exists no longer, but there is a room for receiving the babies from the midwives, who take a receipt, by which the child can at any time be reclaimed, though, alas! it rarely is.

Its object is to receive, maintain, and educate illegitimate children until they are able to provide for themselves. It also receives children born at the Maternita or Lying-in Hospital, which is connected with the building, whose mothers die. It also takes new-born babes of legitimate parents who have no means of nourishing them, but these they keep only one year. The children are all baptized with the surname of Degli Innocenti, which accounts for the name occurring so often in the population.

At the time of our visit the hospital contained forty-five children, who seemed lost in the huge space assigned them, and a good number of old women in another part of the building. The expenses are met by legacies administered by a committee.

There are seven sorvegliente or superintendents and four nurses, not counting the peasant women who come in from the surrounding country to take charge of the babes.

There is a special dormitory for the babies, whose little cots, with their blue and white coverings, look pretty. There is a large bed at one end for the woman who has charge of it. There is a table beside each cot, and even in this ward the floor is of brick.

We went into a room where six tiny mites were waiting for the peasant women to take them home to nurse, and where one or two older were standing inside the queer machine such as one sees in old pictures to prevent children falling down and getting into mischief. It is something like a huge lamp shade in wood with a child in the middle instead of a lamp, only in this case the shade reaches to the ground all the way round. Another was lying comfortably asleep on flannel and oilskin, and each and all looked plump and happy.

The huge play-room was like a church in size, with grim old portraits frowning down on the little ones—there was nothing child-like about it. Passing into a second dormitory we found each cot draped with mosquito curtains, and the infirmary was full of little sufferers in swaddling clothes. There is a separate ward for infectious cases. It is only within the last three years that religious sisters were placed in charge. In the refectory, where the older children and old people and sisters dine together, the tables were neatly laid, and we waited to see dinner served. All entered in an orderly manner, sang their grace, and began on good soup, meat and vegetables following. The assistants to the sisters are, as a rule, those who have been brought up here.

The whole institution, which is perfectly bewildering to a stranger, so many are the staircases and passages, is lighted by oil lamps. As in all old hospitals, the sanitary arrangements are very imperfect.

### Entertainments at the Hospitals.

**Chelsea Hospital for Women.**—A pleasant entertainment was given to the patients at this hospital on January 11th, organised by the Ladies' Committee, beginning with a tea at four o'clock and followed by songs from Mr. and Mrs. Scott Gatty and Dr. Eden, and a zither solo by Dr. Giles. Some clever ventriloquism by Mr. Russell was a source of much amusement. A Christmas tree was provided, and presents of warm clothing and other acceptable gifts were distributed to patients and nurses from Dr. and Mrs. Hugh Teuton.

**City Hospital, Edinburgh.**—On January 23rd, on the invitation of the convener and members of the Edinburgh Health Committee, the nurses of the City Hospital were entertained at an "At Home" in the City Chambers. The invitations included the matrons and nurses of the Edinburgh and Leith hospitals, district, and private nursing homes. The guests were received by the Lord Provost and Bailie Pollard, the convener. The matrons present were Miss Sandford, City Hospital; Miss Wade, Scottish Branch Q.V.J.I.N.; Miss Grant, Royal Scottish Institution; Miss Beveridge, Incurable Hospital; Miss Bourne, Chalmers Hospital; Miss Pigott, Royal Sick Children's Hospital; Miss Guy, Victoria Hospital; Miss Mary Peter, Craig House; Mrs. Sinclair, Belvedere, Glasgow. Many nurses from these institutions were present, and the Royal Infirmary and Leith Hospital were likewise well represented. The scene was very bright and pretty; matrons and nurses were in their distinctive uniforms. At eight o'clock a comedietta entitled "Women's Wrongs" was performed in the Council Chambers, followed by music, and the entertainment ended by a short dance. Many of the leading physicians and surgeons were present, besides a large number of other guests. A most enjoyable evening was spent. This is the first occasion on which the nurses of the different institutions in Edinburgh have been asked to meet in this way, and they hope it may be the forerunner of many such pleasant reunions.

### The Royal British Nurses' Association and Nurse Barlow.

### IMPORTANT STATEMENT BY H.R.H. PRINCESS CHRISTIAN.

By command of Her Royal Highness the President (Princess Christian), a special general meeting of the members of this Association was held on Tuesday at 20, Hanover Square. Sir J. Crichton-Browne presided, supported by Sir J. Russell Reynolds (President of the Royal College of Physicians), Sir W. Broadbent, Mr. Fardon (medical honorary secretary), Miss Wedgwood, Miss Alice Ravenhill (secretary), and several ladies and gentlemen. There was a very large attendance of members of the corporation, every seat being occupied as well as all the available standing room.

Sir J. Crichton-Browne, in opening the proceedings, said he was desired by Her Royal Highness the President to occupy the chair that afternoon and to express to them her regret at her inability to be present. Her Royal Highness was really detained at Osborne by tender ministrations akin to those of a nurse, for she was engaged there in comforting her widowed sister and in sustaining her Royal mother under the heavy bereavement which had fallen on her and cast a shadow over the whole country. He was sure the Royal British Nurses' Association would feel deep sympathy with the Royal Family in their sorrow, and especially with their President, who must have had many anxious hours of late about her own brave soldier son, who had been exposed to exactly the same dangers as those to which Prince Henry of Battenberg had succumbed. It was to be hoped that Prince Victor would return speedily and safely. (Hear, hear.) He then called on the medical hon. secretary to read the notice calling the meeting. But before Mr. Fardon did so he had to request all who might be present but who might not be members of the Association to withdraw, as this was purely a domestic matter for the Association, and strangers could not be admitted.

Dr. Bedford Fenwick, rising from the body of the hall, asked if he should be in order in proposing that Mr. Fowler be allowed to be present to represent Miss Barlow?

The Chairman replied that Miss Barlow would be perfectly safe in the hands of her fellow members of the Association. As he had said, it was a domestic matter, and they did not contemplate further legal proceedings he hoped. He had, therefore, to request that all who were not members of the Association would withdraw.

Dr. Hugh Wood asked, as a mere matter of justice, that Miss Barlow, concerning whom a condemnatory resolution had been sent out all over the world without the facts of her case being stated to those who received the resolution, should be permitted to have her lawyer present to watch the proceedings on her behalf.

The Chairman remarked that he expected Miss Barlow was represented by many friends present. He had ruled, acting on good advice, that those who were not members of the Association must withdraw, and he must insist on their doing so.

Mr. Fardon, having read the notice convening the meeting, said the business on the agenda was to call attention to the action brought by Miss Barlow against the Association, and to move the following resolution:—

"That this meeting is of opinion that the conduct of Miss E. G. Barlow, a member of the Royal British Nurses' Association, in writing a letter complaining of the management of the Association to a public newspaper without previously communicating with the Executive Committee, or bringing her grievance before the proper authorities, and in subsequently bringing an action at law against the corporation and its officers, was disloyal and unjustifiable; and it further desires to record its strong disapproval of any member of the Association resorting to litigation with the corporation, or inciting or encouraging a member to do so, until every possible means of settling the matter in dispute within the corporation itself has been tried."

He remarked that the object of the meeting was not to make a personal attack on Miss Barlow. ("Oh!") To do so would be unworthy of their important corporation, and he wished to make that point perfectly clear at the outset. To what extent Miss Barlow might have been the real and *bona fide* plaintiff in the action which had culminated in this meeting, or whether or not she might have been the instrument of others—(hear, hear)—was a point on which more light might be thrown in the course of that afternoon's discussion. But, however that might be, Miss Barlow's name was used in all the proceedings that had taken place, and it was her name which was appended to all the letters and communications which had reached the Executive Committee directly and through the Press. Therefore the committee had no option in speaking of the plaintiffs in the case against the corporation but to speak of Miss Barlow. He believed that Miss Barlow was a nurse in the employ of Mrs. Bedford Fenwick, and it was not until June 7th of last year that her name came up for membership. She was duly elected, and all who had taken any pains to acquaint themselves with the details of the case knew that, within a very short time after her election, a letter appeared in a public newspaper, signed by Miss Barlow, in which she made serious reflections on the management of the Association, which the Executive Committee considered to be an unjustifiable attack. He was not going into the question whether or not the complaint of Miss Barlow was reasonable or unreasonable. That was not the point. [A voice: "That is the point."] What the Executive Committee said was that if any member of the Association, who was bound by the declaration that she signed to obey the rules and regulations and generally to promote the interests of the corporation, had any complaint to make, she was, in honour and loyalty to the Association, bound to bring it first before the proper authorities, and not to publish it in a public newspaper, and so bring discredit on the Association. (Cheers.) Miss Barlow's action was also unjustifiable because she had never, by bringing her grievance to the knowledge of the proper authorities, sought a remedy for it within the corporation. The Executive Committee, after due deliberation, were specially summoned to consider this case, and they determined to deal with it under the disciplinary powers conferred on them by the bye-law, which says that it shall be in the power of the Executive Committee to summon before them any registered nurse whose conduct, in their opinion, it might be considered necessary to take some sort of notice of or proceedings against, and it also gave further powers to the effect that in the event of the Executive Committee considering it necessary they might direct her name to be erased from the register subject —and this was an important point—to a right of appeal by the nurse to the whole General Council of the Association. It was decided under this special clause to summon Miss Barlow before the Executive Committee, and Miss Barlow was informed of this decision. This led to a good deal of correspondence as to whether or not it was intended to erase her name from the register, and at last the committee got a letter from the solicitors who were advising Miss Barlow, who said they must be informed "Yes" or "No" on the point. That was a reasonable request. Miss Barlow was entitled to know whether or not the Executive Committee was going to proceed against her with the view of striking her name off the register. But just look at the way it was served on them. The letter was received at half-past eleven on the morning of the very day when the annual meeting was being held in the Langham Hall, when all the officers were busy, and the President was momentarily expected. At such a time their secretary was suddenly served with a notice that unless a reply was received in two and a-half hours an injunction would be applied for. It was impossible at such a short notice to give a definite reply. The Executive Committee did the best thing they could, and consulted without delay eminent counsel, Mr. Muir Mackenzie, who said he would do what he could, and he wrote a letter, in which he stated that although he had no authority to give any definite answer he would undertake to attend the proposed meeting and advise the meeting to take no course likely to prejudice their client, and he added that he had no doubt the meeting would act on the advice he tendered. There was no earthly doubt that Mr. Muir Mackenzie's advice would have been followed by the committee, and there was no danger of the nurse's name being struck off the register. He had now laid before them briefly the salient points of the case, and those who had anything to say respecting it would no doubt have the opportunity that afternoon to do so.

The Chairman said he had had just put into his hands a letter from Sir James Paget, who was obliged to keep at home by a cold and cough, but he stated that if he had been able to be present he would have heartily supported the resolution of which notice had been given in the agenda. In a letter received from Miss Bridges, of the Borough Hospital, which was also read by the chairman, the writer said she most thoroughly disapproved of the way the Barlow case had been conducted by the Association.

Sir J. Russell Reynolds, in proposing the resolution appearing on the agenda, and read by the medical hon. secretary, remarked that for an institution like theirs, so young in its history and so promising, apparently, in its prospects, to have been brought to the necessity of summoning such a meeting as the present was a great distress to him personally, and he thought it must be also to those who had its best interests at heart. (Cheers.) He believed the nurses would agree with him, as he was sure the doctors would, that the first thing they had to consider was the care of their patients, and the next thing was, he would not say themselves, but, if they happened to be associated with an institution or corporation like this, the next thing they had to consider was the good of the corporation. (Hear, hear.) It could not be denied that their own individual interests were largely identified with the well-being of the corporation. All these interests by such a fracas as the present must be damaged, and the value of the institution thrown back—for how long he could not say. The confidence of persons was broken in applying to the institution for relief, the Association itself was damaged, and the nurses themselves were, he thought, irretrievably damaged by being mixed up with such a ridiculous quarrel as that in which they were now engaged—merely a storm in a tea-cup. He did not think it possible for any institution or association of persons doing good to others, or any club, to pass over an act like the one now before them. (Cheers.) All he could say was that such an institution or club would not last for a year if persons, because some little matter of detail was not observed, were to write a day or two after they were elected to the public newspapers about it. Imagine, for instance, a person being admitted into the Athenæum, and, because he did not get a notice of something, applying to a lawyer to give him advice about it without consulting the committee of the club. (Laughter.) He did not know what would become of society if such things went on. (Laughter.) We should be broken up into a mixed set of small folks who had forgotten what the objects of the Association were to which we belonged, and thought only of ourselves—the last, lowest, and most contemptible thing a person could take into consideration. He besought all nurses who had the interest of the corporation at heart to forget all their quarrels, and go on to something higher and nobler—something which would be for the advantage of their patients and of the Association. If they were going to continue to squabble about little matters, the value of the Association must be destroyed. He appealed to them, therefore, to be loyal to the Association and to one another, and to the highest interests of the patients who might be committed to their care, and loyal also to their Royal President. (Cheers.)

Sir W. Broadbent, in seconding the resolution, said the only object that he and most of the physicians and surgeons around him could have in attending that meeting was to promote the interests of the nurses and of that great nursing association to which they all in different degrees belonged, and they also desired to promote that cordial co-operation between medical men and nurses which was so necessary for the well-being of patients. (Cheers.) He fully shared the feelings of pain felt by Sir Russell Reynolds in having to attend such a meeting as the present one. The resolution, which he seconded with all his heart, expressed a plain truism. No one in the abstract could contend that the conduct of any member of an Association like this in writing a letter complaining of the management in a public newspaper without previously communicating with the secretary or taking the means open to her to obtain the redress of her grievance by the Association itself—no one would contend that such conduct was anything but factious and wrong. (Cheers.) That it was foolish also had been distinctly shown by the President of the College of Physicians. To his mind the worst feature in the whole case was that it revealed the existence within the corporation of some sort of faction. (Hear, hear.) Certainly everybody could not be working loyally for the Association when proceedings such as had been alluded to by the hon. secretary had been possible. They could not but see that the nurse whose name had been forced into such unwelcome prominence was the instrument of some faction within the Association, the end of which, if continued, could not be otherwise than the destruction of the Association as an association for the public good, for the good of the nurses, and for promoting co-operation between the medical profession and nurses. He had no time to say more, but he had said sufficient to indicate his view of the case now before them. (Cheers.)

Dr. Bedford Fenwick remarked that a more simple letter than that written by Miss Barlow to the newspaper, and about which so much had been said, it was almost impossible to conceive. That letter appeared because of the suggestion that faith was being broken with the ladies who founded the Association. (Hear, hear.) Those ladies were promised a permanent seat on the governing body of the Association, but when the charter was obtained the bye-laws were altered so that those ladies had to retire like other members of the governing body. When that state of things was discovered honourable people advocated that a meeting should be called and the bye-laws altered so as to keep faith with the founders of the Association. The requisition for such a meeting was sent in, but the Executive Committee postponed calling it until it was too late to hold it in order to carry out the object in view. As the bye-laws could not be altered the ladies in question had to retire from the council; but a number of members wishing to do all they could to keep faith with them determined to place their names on the voting papers, and so it became a matter of great importance to members of the Association to obtain a voting-paper. When Miss Barlow became a member of the Association she wished to see faith kept with the founders of it, and so she was anxious to get a voting paper, and it was the fact of her not getting one after she had called at the office for it that caused her to write the letter complained of. He contended that the letter of June 28 to Miss Barlow informing her that the committee had decided to proceed against her in accordance with the powers conferred on them by the bye-law which had been referred to, meant that they had decided to remove her name from the register. ("No!") On receipt of that communication Miss Barlow saw that she must immediately take steps to protect herself if she was not to be professionally ruined, and so she placed the matter in the hands of solicitors, with the result known to them all. In giving judgment, the judge said that the Executive Committee had been most unfortunate in the course they had pursued, and after going through all the details his lordship said, "I very much regret the result, but it seems to me that in these circumstances she (Miss Barlow) is entitled to come to this Court for some relief." The relief that she had obtained was that the threat in the letter of the Executive Committee meant nothing.

The Chairman: No decision was given on the issue. The costs were given against the corporation simply because Miss Barlow was entitled to apply, and the judge, in giving the costs, twice expressed his regret that he was technically compelled to do so.

Dr. Bedford Fenwick, continuing, said the General Council had had no opportunity of expressing its voice on this question, and yet a resolution had been sent out by the Executive Committee condemning Miss Barlow unheard. That was an un-English proceeding, and one that he did not believe the English people with their innate love of justice would tolerate. He especially wished to emphasize the point that Miss Barlow sought the protection of the law only because she was threatened in her professional standing, and he maintained that she was perfectly justified in doing so. (Cheers.)

Dr. Hugh Wood moved as an amendment:—

"That this Association declines to deter its members from using their private judgment as to the propriety or otherwise of publicly criticising the management of the Association in a legitimate way, and further declines to use its influence to prevent its members from exercising their legal right of applying to Her Majesty's courts of justice whenever they think right to do so."

He thought there had probably been fault on both sides in connection with this matter, but, however that might be, he was certainly of opinion that the executive had acted in a high-handed manner.

Miss Beatty seconded the amendment.

Mr. Biernacki thought it was time they stopped this miserable discussion. Every member of the Association who had gone into the case must feel that mistakes had been made on both sides. Miss Barlow's mistake was in writing an indiscreet letter to a newspaper, but he thought that that letter would have been very much better met by a dignified statement of the facts by the other side in the next issue of the journal. It appeared to him that the time had now arrived for dropping this question altogether, and he thought it would be possible to frame an amendment which would cast no reflection on either party. He had seen a reference to this meeting in a certain paper as "the next stage." He thought there should be no further stage, and he moved:—

"That Miss Barlow's action in writing to a public newspaper with reference to the non-delivery of voting forms is not a matter of sufficient importance to call for a formal condemnation on the part of this meeting of the Royal British Nurses' Association; and, further, that it is the opinion of this meeting that Miss Barlow brought her action against certain members of the Association in the absence of definite information on a point which greatly affected her interests."

Dr. LOVELL DRAGE said the amendment just moved so fully expressed his views that he had much pleasure in seconding it. He had hoped that Sir Russell Reynolds would have stopped, so that they might have had the opportunity of prevailing on him to withdraw his resolution. All of them who felt a great interest in the affairs of the corporation must share in Sir Russell Reynolds' expression of regret at the present unfortunate circumstances. This was not the first time there had been discord in the Association, but he felt sure that he should carry the meeting with him when he said it must be the last. (Cheers.) It must be the last, because if these perpetual frictions were to be kept up the corporation would end in total failure. (Cheers.)

Miss WEDGWOOD said that, as a nurse who was trained in two great London hospitals, the methods employed by Miss Barlow in connection with late events seemed to her incomprehensible. Not the least of the advantages of a long stay in great hospitals was the opportunity of gaining the habit of discipline, obedience, and dutiful loyalty to authority. (Cheers.) Without these qualities a nurse could scarcely be useful, and she might be very dangerous. She did not mean for a moment that anybody should ignominiously submit to injustice, but she did mean that they were all bound to be loyal to any association to which they belonged, and that one of their first duties if wronged was to try and obtain redress from within the Association. She supposed that as they had all promised to maintain the dignity of the corporation, they ought all to testify their loyalty to it and to their Royal President, and their gratitude to those distinguished men who gave them freely of their time and substance to help the members of a profession which, although closely allied to their own, was, and always must be, distinctly subordinate to it. (Cheers.)

Dr. NORMAN KERR thought it probable that both parties were partly right and partly wrong, and if that view were taken by the majority it might be possible for the Executive Committee to see their way to withdraw the original resolution for one which he would submit.

The CHAIRMAN, intervening, said the executive could not depart from their resolution, which had received the support of Her Royal Highness the President.

Dr. NORMAN KERR remarked that he would then propose, as a fresh amendment, the following:—

"That this meeting desires to record its strong disapproval of any member of the Association resorting to litigation with the corporation, or inciting or encouraging a member to do so, until every possible means of settling the matter in dispute within the corporation itself has been tried."

The object he had in proposing that was that they would thereby, in a very courteous way, and without pronouncing a strong opinion on either side, be able to record their strong opinion of the indiscretion that had been committed.

Dr. HEYWOOD SMITH, in seconding the amendment, stated that when he was honoured by a place on the council of the Association he took every means he could to inform himself of the manner in which the work of the Association was done. He had been able to satisfy himself that the work was well done, and he must express his strongest confidence in the executive. He did not wish to say anything about the case they had met to discuss, except to ask, Who were the people that inspired the nurse to do the things of which complaint had been made? (Cheers.) Miss Barlow happened to be a nurse who was employed in a private nursing establishment belonging to the editor of the *Nursing Record*, and whose husband they had had the pleasure of hearing on so many recent occasions. (Laughter.) He hoped, if it were possible, Dr. Norman Kerr's proposal would be accepted.

Mr. B. CARTER explained that what happened when Miss Barlow called at the office for a voting paper was this. She was told by the secretary that the voting papers had been sent out some time ago, and that it was not the custom to issue voting papers to new members after the bulk of them had been sent out; but the secretary said that, although she did not feel authorized to give Miss Barlow one, she would ask for authority to do so. The next day the secretary received the necessary authority, and the voting paper was sent to Miss Barlow.

Mrs. BEDFORD FENWICK said that during the past two years a new form of government—one of official autocracy—had been introduced into the Association. Dr. Drage had stated that there had been friction in the Association before. She could only say that there would continue to be friction until there was justice shown towards the nurses. (Hear, hear, and hisses.) No one could have more truly at heart the interests of the Association than herself, but she did not intend to be content unless it was conducted on proper lines. They must not imagine that Miss Barlow was the only one who felt herself aggrieved by the present management of the Association. Her contention was that the basis of the corporation must be justice for the nurses, who must be permitted to express their views and wishes in their own Association. (Cheers.)

Miss THOROLD cordially supported the resolution proposed by Sir Russell Reynolds, and appealed to her fellow-nurses to vote for it.

Miss KENEALY complained that the register was being used by the Executive Committee as a weapon to prevent the nurses exercising their right of criticising the management of their own Association. She had the misfortune to be on the General Council of the Association for three years, and her experience was that whenever she tried to bring forward any matter every means was tried by the chairman for the time being to stifle discussion. In fact she had no hesitation in saying that every time she tried to do her duty towards the nurses during those three years she had been snubbed.

Dr. BEZLEY THORNE argued that the letter of Miss Barlow to a certain newspaper contained statements that were untrue, and the proper course for her when they were pointed out was to withdraw it publicly. He repeated that the letter was untrue.

Dr. BEDFORD FENWICK: Prove it.

Dr. THORNE: It has been proved.

Dr. BEDFORD FENWICK: Not to us.

Dr. THORNE said it had been represented that the letter was a complaint against the Executive Committee. On the contrary, it was an attack on the individual, which made it the more despicable. It had further been represented that the answer which was sent by the honorary officers was a threat to erase the name of Miss Barlow from the register. That was quite untrue, and it was inconceivable to him how anyone could have the hardihood to make the assertion. It was evident from the bye-law that when the Executive Committee took action under it the first part of their proceeding was to inquire and give the persons against whom the proceedings were taken an opportunity for making a full reply. The only threat levelled against Miss Barlow was that she would be required to appear before the Executive Committee and explain what she meant. In view of all that had taken place he had come to the conclusion that neither the nurse who wrote the letter nor those who stood behind her were prepared to face an inquiry. (Cheers.)

Miss DE PLEDGE said she strongly approved of the resolution of the Executive Committee, and she desired to support it, because it was a matter of discipline.

Miss POE stated that as a nurse member she was forbidden at the last council meeting to speak; in fact, discussion was stifled. She asked that nurses might have the right on all occasions when they found it necessary to write to their professional newspapers as freely as doctors wrote to the medical press. (Cheers.)

### Statement by H.R.H. Princess Christian.

The Chairman then read the following message from Princess Christian, the President, dated Osborne, January 26th: "You are all aware of the grave matters which have for some months seriously threatened the best interests of our Association. As President, I have thought it right to summon a special general meeting to consider the causes of this trouble, which had its origin in a letter from Nurse Barlow gravely reflecting upon the officers of the corporation, and which has led to an action at law. I think the time has come when all the members should be made fully acquainted with recent events, and I feel confident that with a full knowledge of these the nurses will accord their complete approval of the action of their officers. I would beg you to weigh the facts well, to realise the grave importance of your decision, and that as members of a corporate body it is in your power to forward or injure lastingly the interests of your cause. I cannot conceal my disapproval or the pain I experience at the disloyal course adopted by Nurse Barlow and her advisers, which cannot fail seriously to cripple the resources of the Association and divert them from their intended uses. I would also like to remind you of the time and strength given not only by myself but by all the officers, loyally, ungrudgingly, and unceasingly, and given at much individual cost for no individual advantage or gain, and with no other motive than the welfare and prosperity of nurses. The management of the Association is confided to the hands of a few, and in a large body such as ours is there must be necessarily a divergence of opinions, but individual interests and grievances should always be subservient to the general good. We can never prosper unless the fullest confidence and trust are placed in the acting officers, who ought to be upheld and supported in their arduous exercises and labours. Unity is strength, and I feel certain that if you will all work loyally with me and those who devote their best energies to the great and good work of the Association, you will help to consolidate it, to increase its prosperity, and thereby to accomplish the objects for which the Association was founded and the charter granted." (Cheers.)

Dr. Hugh Wood having withdrawn his amendment, the two remaining amendments, moved by Dr. Norman Kerr and Mr. Biernacki respectively, were submitted to the meeting, and were both negatived. The original motion was then put, and declared to be carried by a large majority.

Dr. Bedford Fenwick called for the names of those members who voted for and against the resolution.

The Chairman replied that that was a question for the meeting to decide.

A Member asked if the charter specified any particular way of taking the vote.

The Chairman said it did not.

The meeting having expressed itself satisfied with the result of the voting on a show of hands, the names of the voters were not taken.

On the motion of Mr. B. Carter, a vote of thanks was accorded to the chairman for presiding.

The Chairman, in acknowledging the compliment, asked those present to give some expression of their devotion and loyalty to their Royal President. (Cheers.) He felt certain, notwithstanding the remarks they had heard during the discussion, that the Association would now enter on a course of peace, prosperity, and usefulness.

The proceedings then terminated.

### Wants and Workers.

[The attention of correspondents is directed to the fact that "Helps in Sickness and to Health" (Scientific Press, 428, Strand) will enable them promptly to find the most suitable accommodation for difficult or special cases.]

Will J. E. S., stamp collector, kindly write to M. K., 4, Fairbridge Road, Upper Holloway, N.?

### The "Oriolet" Hospital.

### A HOSPITAL FOR VEGETARIANS.

The latest addition to the ranks of special hospitals is probably one which early in last year was started by certain ardent believers in the gospel of vegetarianism on the borders of Epping Forest, at Loughton, in Essex. Here in pretty grounds stands a somewhat quaint-looking house, Oriolet by name, and this has been selected by Mr. Arnold F. Hills and Mr. Josiah Oldfield as suitable for a dietetic experiment on certain diseases, and endowed by the former for three years with a definite sum. By way of further support a sustentation fund is being raised for the purpose of paying the fees of very poor patients when their first fortnight, for which admission is free, is over. For patients who can pay for themselves the fees range from 10s. to £1 a week.

Cases of cancer are made the first consideration, it being a special ambition of the promoters of the institution to observe the effect of vegetable diet on this particular disease. Patients on entering the home are, of course, expected to submit to a purely vegetarian diet, and as a rule find little to complain of in the change of nourishment. The nursing staff themselves are enthusiastic abstainers from flesh foods, from the matron (Miss Gertrude Hick) to the one probationer. It is a somewhat amusing fact, however, that the visiting medical officer is a notable exception to this universal rule. Miss Hick was trained at the London Hospital, and as the wearer of a particularly picturesque red uniform drew some special attention at the Royal National Pension Fund Garden Party at Marlborough House in the summer.

Patients of both sexes are received at Oriolet. The wards are sunny and bright, "sun baths" being a feature in the treatment. In addition to the general wards (the men's named "Orœa," the women's "Pandora") there are private rooms for the use of paying patients. As mentioned above, certain cases are received free of charge for the first fortnight at least, and those suffering from cancer may, under certain conditions, remain free of charge for three or six months.

The number of patients has been gradually increasing, the treatment being stated to prove very satisfactory, and a careful record is kept of the cases with their results. Mr. Oldfield gives himself to the service of the cause he has at heart, and is the warden of the home, over which he spends much time and thought. A little chapel forms part of the equipment, where services are held by the honorary chaplain, the vicar of one of the Loughton churches.

Speaking from an unconverted point of view, it is to be noted, with congratulations to the patients and inmates generally of Oriolet, that milk and eggs are not forbidden articles of diet. This is not, of course, tolerated by the straightest sect of the vegetarian community, but enters into the dietary of the Essex home, and no doubt helps much to reconcile patients to a more or less unfamiliar system of feeding.

Help in the way of old linen, under garments, and gifts in kind, would be very thankfully received by the matron; or new calico and other materials for the patients to make into clothes for themselves.

### Appointments.

Brighton, Hove, and Preston Dispensary.—Miss Maude Grey has been appointed Matron of this institution. She received her training at the Sussex County Hospital, and has since held the post of head nurse at the Western Branch of the Dispensary in Brighton.

North-Eastern Hospital, Tottenham.—Miss Ellen Buxton has been appointed superintendent of nurses at this hospital. She was trained by St. John's House at Charing Cross Hospital, Metropolitan Hospital, Kingsland Road, Soho Hospital for Women (three years' inclusive training and certificate). Miss Buxton then did private nursing on St. John's House Staff for a year and a half, and subsequently held the post of charge nurse at the North-Eastern Hospital for two years, afterwards working for three months in the Riverside Hospital, New York. We wish Miss Buxton every success in the hospital, where she is already well known and valued.

### Everybody's Opinion.

[Correspondence on all subjects is invited, but we cannot in any way be responsible for the opinions expressed by our correspondents. No communications can be entertained if the name and address of the correspondent is not given, or unless one side of the paper only be written on.]

### NURSES FOR PRISONERS.

Nurse Maud writes: I shall be obliged if you can obtain some information about prison nursing for me. I am a trained nurse of some years' experience, and I should like to know whether prison work is worth taking up? Should I find a good field of work, and is it such as a trained nurse could follow without losing her standing in the nursing world? Where could I learn particulars?

[We shall be glad to have letters on this subject, and definite information as to where trained nurses are already employed in prison hospitals. We do not understand our correspondent's question as to whether this work is "worth taking up." Surely that depends entirely on her own motives in approaching it, and still more on the spirit in which she goes in for it. How can a nurse lose her "standing" by doing good work conscientiously, whether it be tending a convict or the judge who condemned him? It seems to us that only a limited number of women will ever be found willing to take up prison work. They will need to be women of the best sort—high principled, courageous women, who enter upon the work (which cannot fail to be trying) for the sake of sick and suffering humanity, not for their own benefit. We hope some of our readers will write to us on the subject of "Trained Nurses in Prisons."—Ed. *T. H.*]

### NURSES AND LAY COMMITTEES.

"A Superintendent of District Nurses" writes: May a district matron suggest that as many rich people decide for themselves that they will have the attendance of a trained nurse, it is not unreasonable that the poor should be allowed the same liberty when a nurse is to be had? It does not appear that there can be any more objection to a lay committee for the management of a district nursing association than of a hospital; and, provided that only thoroughly trained nurses are employed, there ought not to be any difficulty, as a properly trained district nurse would naturally, when able to do so, communicate with the medical man in attendance on a case, and he may be asked to visit and receive his directions—quite regardless as to whether he "continues his subscription or not." There must, nevertheless, always be—both in town and country districts—a number of chronic cases greatly needing the care that only a trained nurse will give; and surely no one will say that it is not right for these to be attended, and bed-sores prevented, even though they may only be visited occasionally by a medical man, and no special treatment is required. It can hardly be expected that a busy doctor can always stop to ascertain (even if he wished to do so) which of the people he finds in the houses of his poorer patients are responsible for the attendance given to these chronic cases; and if the relatives know that they can have the help of a nurse, it is natural they should try to get it. And if the nurse does her best to carry out the general directions given to the friends it does not seem fair to complain of a system which provides this help for the poor, whose difficulties in sickness are so great. It is certainly the rule that medical men accept readily the services of competent district nurses, but it is equally certain that those nurses (?) who are inexperienced and fussy are little likely to be acceptable to doctors, patients, or their friends. The "radical change" that "Criterion" deems necessary in district nursing will doubtless be accomplished in time, when people have been converted from the belief that "any kind of nurse will do for district work," and will cease to expect any other service but that of attendance on the sick from a district nurse.

### Notes and Queries.

The contents of the Editor's Letter-box have now reached such unwieldy proportions that it has become necessary to establish a hard and fast rule regarding Answers to Correspondents. In future, all questions requiring replies will continue to be answered in this column without any fee. If an answer is required by letter, a fee of half-a-crown must be enclosed with the note containing the enquiry. We are always pleased to help our numerous correspondents to the fullest extent, and we can trust them to sympathise in the overwhelming amount of writing which makes the new rules a necessity. Every communication must be accompanied by the writer's name and address, otherwise it will receive no attention.

### Queries.

(123) *Address.*—Can you give me the address of Miss Le Pelly, who, I think, is connected with a village nurse association?—*A. M. A.*

(124) *Co-operation.*—Please tell me where the Co-operation of Nurses is situated in London, and what is the Registered Nurses' Society.—*Sister.*

(125) *Co-operative Principle.*—I have found out that the Registered Nurses' Society is a private nursing association, but can you tell me if it is on the co-operative principle, and what is the address?—*A. D.*

(126) *Massage.*—Kindly tell me of any association where I can get a certificate for proficiency in massage.—*Trained Nurse.*

(127) *Training.*—Where do you advise young women of twenty-three to enter for training, &c.?—*G. E. R.*

(128) *Certificate.*—I shall be greatly obliged if you will kindly give me the information I ask. A friend of mine entered an infirmary as a probationer-nurse, and after eighteen months was sent away by the lady superintendent, and told that there was not the least fault to be found in her character and conduct, but that she was "inefficient" and "unsuitable." The lady superintendent refused to give my friend a testimonial of nursing, or any written testimonial. Is she entitled to a written certificate of any kind? And can she demand a written certificate of character?—*Agnes.*

(129) *Lady Managers.*—Can you tell me if any hospitals have ladies on their boards of management?—*A. M. P.*

(130) *Uniform.*—Do workhouse infirmary nurses wear outdoor uniform? Why do not all general infirmaries adopt the same uniform?—*One in Doubt.*

(131) *Untrained.*—Can you tell me the address of some private nursing home where a probationer would be received for a small premium? I am twenty years of age, and not quite strong enough for training in a general hospital?—*Frances.*

(132) *Prevention of Epidemics.*—Can you tell me where and when the statements by Mr. Francis Vacher were made, which were referred to in the annotation in The Hospital of January 4th, headed as above?—*E. R.*

(133) *Intemperance.*—Can you recommend a really good medical book on intemperance? One was reviewed about two years ago in The Hospital, but I cannot remember author's name.—*Torquay.*

### Answers.

(123) *Address (A. M. A.).*—If you write to Miss Le Pelly, care of "Nursing" Editor of The Hospital, we will forward your letter.

(124) *Co-operation (Sister).*—The Nurses' Co-operation is at 8, New Cavendish Street, and Miss K. Philippa Hicks is the lady superintendent. You can get information about the Registered Nurses' Society from Mrs. Bedford Fenwick, 20, Upper Wimpole Street.

(125) *Co-operative Principle (A. D.).*—The address of Mrs. Bedford Fenwick is 20, Upper Wimpole Street, London, and she will no doubt answer the other part of your question if you apply to her.

(126) *Massage (Trained Nurse).*—Write your questions to Mrs. Arthur, Hon. Secretary, Society of Trained Masseuses, 12, Buckingham Street, Strand, and enclose stamped envelope for reply.

(127) *Training (G. E. R.).*—Write to a large hospital where nurses are trained, and ask the matron for a copy of rules, &c. Twenty-five is the lowest age at which probationers are received at some hospitals. You speak of "influence," but that is not required. A suitable woman is accepted on her own merits, but she has to wait for a vacancy, of course.

(128) *Certificate (Agnes).*—If your friend is inefficient and unsuitable for a nurse she is not entitled to a certificate. She certainly cannot "demand" a certificate of character; but should she be in treaty for a situation (which she may be, perhaps, better fitted for) we do not imagine that the lady superintendent you refer to would refuse to answer questions as to character and conduct.

(129) *Lady Managers (A. M. P.).*—We fancy not. Many have ladies' committees, who report to, and work in connection with, the board of managers. Mixed committees would certainly have many advantages if the right sort of women could be elected.

(130) *Uniform (One in Doubt).*—Not always. Why should the nurses of all infirmaries or hospitals wear the same uniform? It would be neither acceptable nor advisable. The reputation of the various training schools varies as much as the colours of the probationers' gowns.

(131) *Untrained (Frances).*—We have often pointed out that it is unwise to begin training as a nurse under twenty-one years of age, and if you mean to follow nursing as a profession it would be of little use to you to enter any private nursing home. If you are strong enough for nursing at all, why not enter some provincial children's hospital as a first step? Get "How to Become a Nurse" from the Scientific Press, 428, Strand, which will give you full particulars of the various institutions to which you might apply.

(132) *Prevention of Epidemics (E. R.).*—Mr. Francis Vacher's statements were made in a presidential address delivered to the Society of Medical Officers of Health. The address is published, and may, no doubt, be obtained direct from Mr. Vacher (Liverpool), or from his publishers.

(133) *Intemperance (Torquay).*—We cannot trace the book you mention unless you can give the date when the review appeared. You might get "Inebriety or Narcomania," by Norman Kerr, published by H. K. Lewis, 136, Gower Street, W.C., price 21s., or either of the following: "Alcoholism and its Treatment," by Dr. John E. Usher (Baillière, Tindall, and Cox, 20, King William Street, W.C.), price 2s. 6d.; or "Disease of Inebriety," published by the American Association for the Study of Inebriety, which can be obtained from John Knight and Co., Bristol, price 6s.

### For Reading to the Sick.

### SUFFERING.

**Motto.**

What else could knit

You theirs, but sorrow? —*R. Browning.*

**Verses.**

With a soul that ever felt the sting
Of sorrow, sorrow is a sacred thing.
—*Cowper.*

Only those are crowned and sainted,
Who with grief have been acquainted,
Making nations nobler, freer!
—*Longfellow.*

Who is the Angel that cometh?
Pain!
Let us arise and go forth to greet him;
Not in vain
Is the summons come for us to meet him;
He will stay
And darken our sun.
He will stay
A desolate night, a weary day,
Since in that shadow our crowns are won!
Let us say still while his bitter chalice
Slowly into our hearts is poured—
"Blessed is he that cometh
In the name of the Lord." —*A. Prbctor.*

**Reading.**

He that is afraid of pain, is afraid of something that will always be in the world; but this is a failure in reverence and respect.—*Marcus Aurelius.*

Thank God, bless God, all ye who suffer not
More grief than ye can weep for.
—*E. B.Browning.*

The sorrows of life are many, and the Saviour made this one of His credentials, that He could transfigure them all into consolation. "The spirit of the Lord God is upon me, because He hath anointed Me to preach good tidings unto the meek; He hath sent Me to bind up the broken-hearted . . . to comfort all that mourn." . . . In that life and that death, in that voice of sympathy and that heart of love, in those sayings and doings of Jesus Christ, which enter into all experience and reach backward and forward into the eternity, above all is that Person, God for us, and God with us. Who manifested to bear our sins and carry our sorrows, on purpose that we might never feel earth lonely nor heaven unreal, has been found through eighteen centuries, is found today, shall be found in the ages to come, a rest and a peace and a satisfaction, which the world can neither give in its joys nor take away in its bereavements. The comfort spoken of is no childish soothing, no effeminate lulling, no palliation of distress, no oblivion of sorrow; it is what its name bespeaks it—a strengthening and fortifying thing, because it both pierces to the depth of the reality that is, and rises to the height of that other reality that shall be.

"The life we live is full of suffering; it is the condition of our existence as the followers of a thorn-crowned chieftain. Pain to be borne, and painful duties to be fulfilled; while, moreover, endurance of the one and fulfilment of the other are wont not unfrequently to produce an effect upon our sensitive, impressionable spiritual being which to some becomes almost a lifelong agony. To the Christian, realising that his special calling is to take up his cross daily and follow his suffering Lord, this will seem no strange thing."—*Sydney Lear.*

### Where to Go.

Alexandra Hospital for Children with Hip Disease, Queen Square.—Christmas treat on Thursday, January 30th, half-past three to six p.m.

National Health Society.—Next Tuesday afternoon's lecture at 53, Berners Street, will be on "Milk and Water," by Mr. Ernest Hart. Amongst the lecturers on following Tuesdays will be Mrs. Scharlieb, Mrs. Clare Goslett, Dr. G. Vivian Poore, Dr. Edward Squire, and Dr. Charles Shelly.

Royal British Nurses' Association. — The second sessional lecture of the season will be given on Friday, January 31st, at eight p.m., at the offices of the Royal British Nurses' Association, 17, Old Cavendish Street, by Mr. Clinton Dent, the subject being "Mountain Myths and Legends," with lantern illustrations. Members are admitted free, the general public on payment of 1s.

Hospital for Epilepsy and Paralysis.—Miss Mathilda Ellis, of 25, Kilburn Park Road, assisted by her friends and pupils, cheered the inmates of this hospital on Monday last with a varied entertainment, comprising songs, recitations, and dramatic duologues, which gave great pleasure to the audience. Miss M. Ellis, Miss M. Hyam, Miss A. Isaacs, and Miss Kezzor contributed to the latter form of entertainment, while the musical and vocal portion included a violin solo by Miss A. Hyman and songs by Miss White, Miss A. White, Miss Simms, and Mr. Richard Hyam.

Cookery Exhibition at the Imperial Institute, from April 27th to May 2nd. Their Royal Highnesses the Duke of Connaught and the Duke of Cambridge are patrons of the undertaking. Every possible result of the kitchen and food products will be represented. The first group is devoted to artistic cookery. The other groups include culinary literature, household cookery, bread and cereals, provisions, dining-room and kitchen accessories. Several new features are promised, among which may be mentioned model kitchens for the mansion and cottage, cookery demonstrations by cooks of the Brigade of Guards stationed in London, the Royal Navy, and the Mercantile Marine Cookery Schools in Great Britain, and practical demonstrations of Indian cookery by native cooks. Further particulars can be obtained from the hon. secretary, C. Herman Senn, 329, Vauxhall Bridge Road, S.W.

St. John Ambulance Lectures at the Polytechnic.—The first of a course of lectures on "First Aid," to be followed by a second course on "Home and Sick Nursing," having been announced for January 23rd (admission free), a fairly large number of ladies put in an appearance at the Polytechnic at a quarter-past three on that day, and a good deal of not unreasonable disappointment was felt when, after a long peroration by Sir Herbert Perrott on the aims of the St. John Amublance Society (with which, it is to be presumed, many present were already familiar), the lecturer, Mr. Andrew Clark, announced that the lecture for that afternoon would not be as advertised. It was proposed instead to explain the general principles upon which the lectures would be given, and to give an outline on the structure of the human skeleton, to be followed by a few remarks on fractures, bleeding, and insensibility. Mr. Clark proceeded to point out the principal bones and joints with the aid of excellent limelight illustrations, going on to speak of fractures, how to distinguish them and treat them, of bleeding and the steps to take to arrest it, concluding with a brief outline of the functions of the heart, the spinal cord, and the brain. A demonstration on bandaging followed, but the lecture on nursing was postponed to the following week. In covering so much ground in the short space of one hour the most sketchy treatment was inevitable, and it is to be feared that those of the audience who do not intend following the whole course will have gone to their homes with somewhat dangerous ideas of what it will behove them to do with umbrellas and brooms, walking-sticks, and handkerchiefs the next time they happen to be within reach of a street accident and a fractured limb. The course of lectures as advertised will now, it is understood, begin on Thursday, January 30th, at a quarter-past three p.m.

